# Supplementary material for: Comparison of deep neural networks to spatio-temporal cortical dynamics of human visual object recognition reveals hierarchical correspondence
Source: Sci Rep. 2016 Jun 10;6:27755. doi: 10.1038/srep27755 (PMC4901271; doi:10.1038/srep27755)
Supplement: Supplementary Information [file srep27755-s1.pdf]

Supplementary Material

Comparison of deep neural networks to spatio-temporal cortical dynamics of human visual object recognition reveals hierarchical correspondence

Radoslaw Martin Cichy[1,2], Aditya Khosla[1], Dimitrios Pantazis[3], Antonio Torralba[1], Aude Oliva[1]

[1] Computer Science and Artificial Intelligence Laboratory, MIT, Cambridge, MA, USA

[2] Department of Education and Psychology, Free University Berlin, Berlin, Germany

[3] McGovern Institute for Brain Research, MIT, Cambridge, MA, USA

Supplementary Figure 1 2
Supplementary Figure 2 3
Supplementary Figure 3 4
Supplementary Figure 4 5
Supplementary Table 1 7
Supplementary Table 2 8
Supplementary Table 3 9
Supplementary Table 4 10
Supplementary Table 5 11
Supplementary Movie 1 12
Supplementary Text 1 13

Supplementary Figure 1

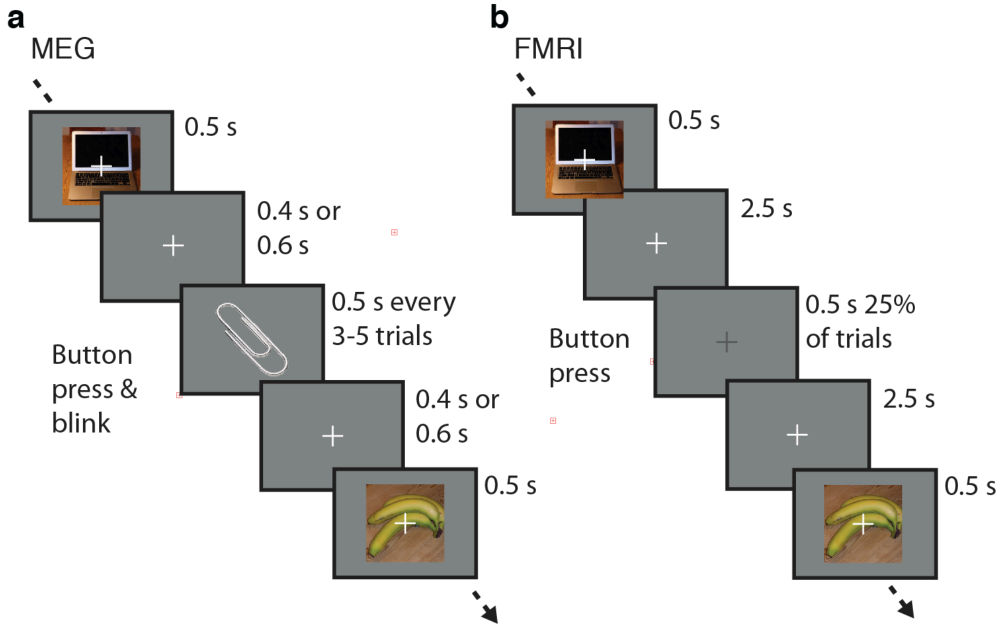

Supplementary Figure 1 Experimental design in MEG and fMRI. Participants viewed the same set of 118 images (4° visual angle, 500ms presentation time) overlaid with a light gray fixation cross. We adapted presentation parameters to MEG and fMRI constraints. **(a)** For MEG, images were presented with an inter-trial-interval (ITI) of 0.9-1.1s. Every 3-5 trials (average 4), a paper clip image was presented prompting participants to press a button, and blink or swallow if necessary. **(b)** For fMRI, the ITI was 3s. The design included null trials (25% of trials) characterized by a change of fixation cross hue to a darker gray and no image presentation. Participants reported null trials with a button press. Object images shown as exemplars are not examples of the original stimulus set due to copyright; the complete stimulus set is visualized at http://brainmodels.csail.mit.edu/images/stimulus_set.png.

Supplementary Figure 2

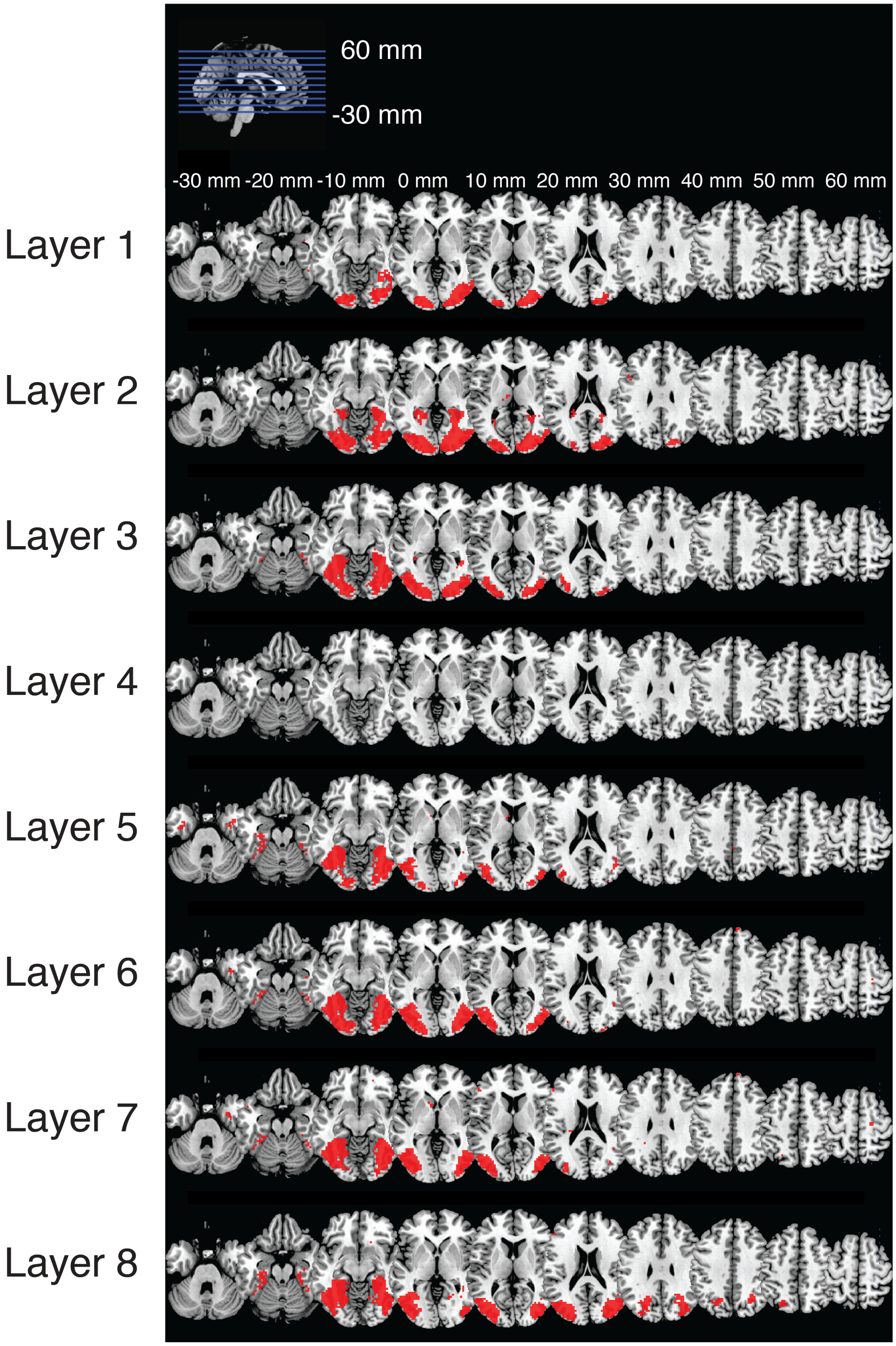

Supplementary Figure 2 Representational similarities between layers of the object DNN and the human brain. Low DNN layers were mapped largely to the occipital lobe of the brain, i.e. low- and mid-level visual regions, whereas high DNN layers to more anterior regions in the temporal and parietal lobe. In particular, representations in DNN layer 8 were found to be similar to brain representations reaching into inferior parietal cortex ($P < 0.05$ by sign-permutation test, $n = 15$, FDR-correction). Each row shows axial cuts positioned in standard MNI space. Overlays were created with MRIcron.

Supplementary Figure 3

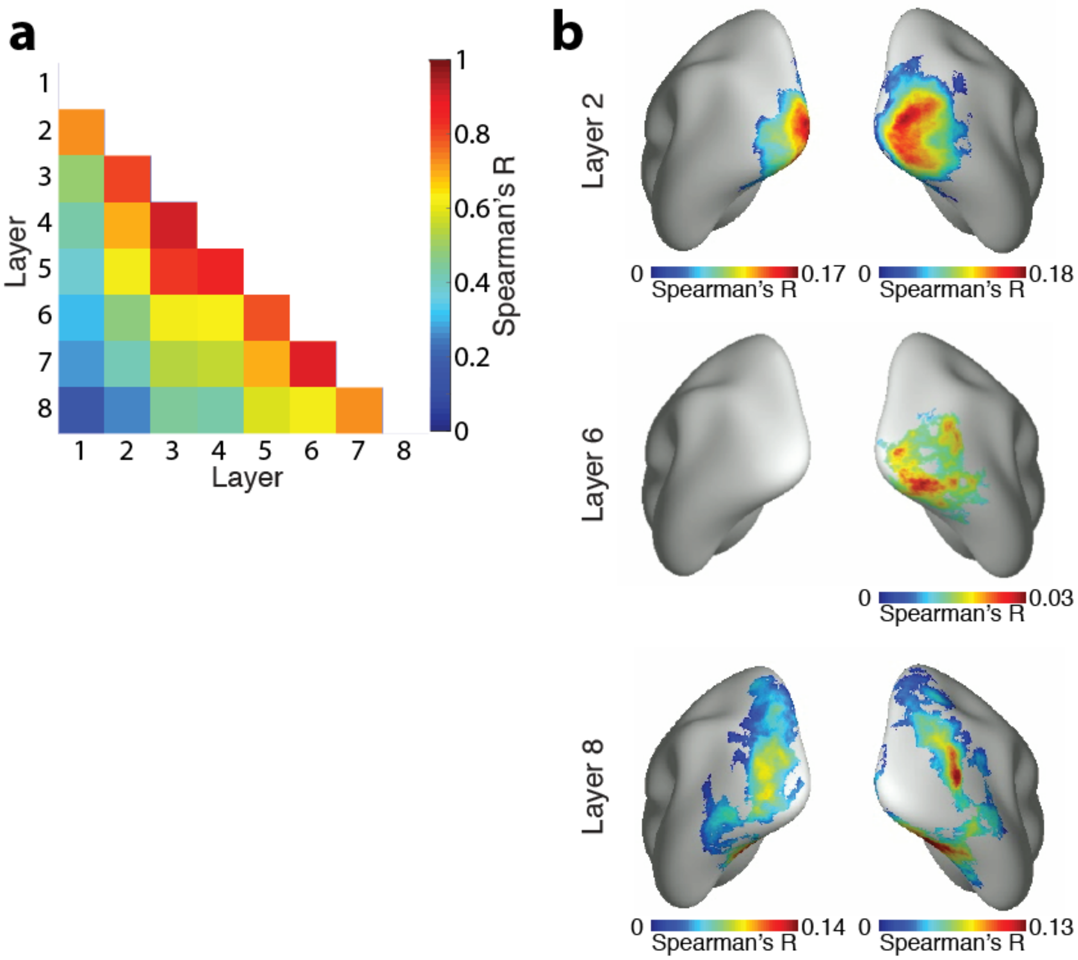

Supplementary Figure 3 Unique contributions of layer-specific DNN RDMs to the fMRI-DNN similarity maps. (**a**) Correlations (Spearman's R) between layer-specific DNN RDMs. Significance was assessed using label-permutation tests (10,000 permutations). All pair-wise correlations were P = 0.0001, and thus significant at the P = 0.05 level, Bonferroni-corrected for pairwise tests ((8*8)-8)/2=28). (**b**) We used partial correlation to furnish spatial maps of visual representations common to brain and object DNN considering variance unique to each layer only, i.e. partialling out the effect of all other DNN layers. We found significant clusters for layers 2, 6 and 8 (n = 15, cluster definition threshold P < 0.05, cluster-threshold P < 0.05 Bonferroni-corrected for multiple comparisons by 16 (8 DNN layers * 2 hemispheres). Corroborating the analysis in Figure 4 at the main article, there was a correspondence between object DNN hierarchy and the hierarchical topography of visual representations in the human brain.

Supplementary Figure 4

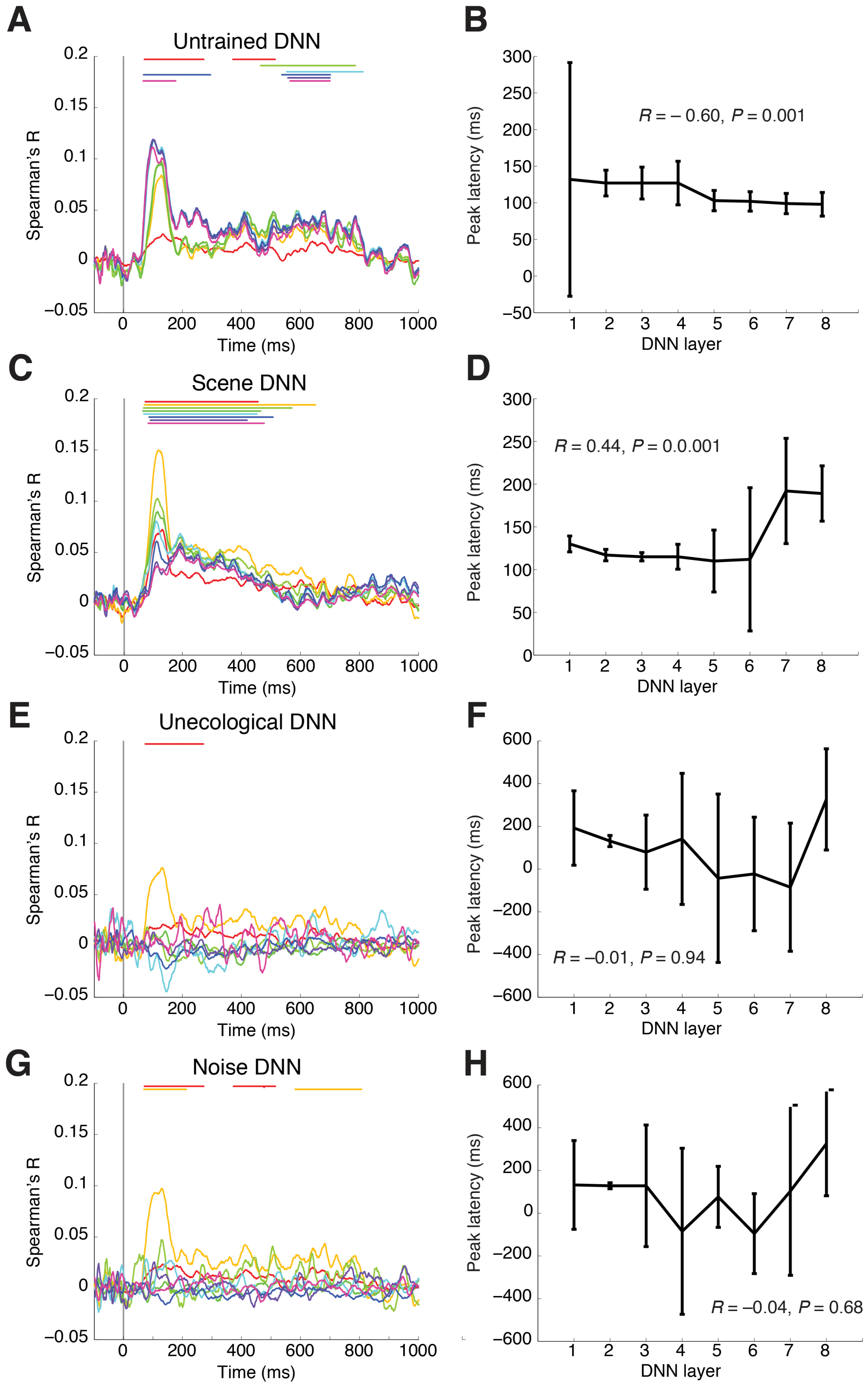

Supplementary Figure 4 Architecture, task constraints, and training procedure influence the DNN's predictability of temporally emerging brain representations – *layer specific analysis*. Representational similarities between layer-specific DNN RDMs and MEG RDMs over time (left panel) as well as the relationship between DNN hierarchy and peak-latency of time course foe ach layer (right panel) for untrained DNN **(a,b)**, scene DNN **(c,d)**, unecological DNN **(e,f)** and noise DNN **(g,h)**. All DNNs had time points with significant common brain-DNN representations for some layers. A significant positive hierarchical relationship between layer number and brain-DNN representational similarities was present in the scene DNN (d, $R = 0.44$, $P = 0.001$), and a negative relationship for the untrained DNN (b, $R = –0.60$, $P = 0.001$). Insets in the right panels show the correlation values between layer number and brain-DNN representational similarities as well as significance (sign-permutation text, 10,000 permutations). In the left panel, lines above data curves indicate significant time points ($n = 15$, sign-permutation tests, cluster definition threshold $P = 0.05$, cluster threshold $P = 0.05$ Bonferroni-corrected by 8, i.e. for number of DNN layers for each panel).

Supplementary Table 1

	Database	
	**SUN-397**	**Caltech-101**
	**Accuracy (% classification)**	
Object DNN	43.45	86.22
Scene DNN	53.10	62.23
Untrained DNN	0.25	0.99
Unecological DNN	0.25	0.99
Noise DNN	0.25	0.99

Supplementary Table 1 DNN performance on scene and object classification. We assessed the performance of the DNNs on object and scene categorization by testing the prediction performance of support vector machines (SVMs) based on DNN layer 7 activations for the scene image dataset SUN-397 [16] and the object-image dataset Caltech-101 separately. In detail, we used liblinear to train one-versus-all SVMs with a linear kernel (L2-regularized L2-loss) for all image classes of each of the image set data sets. We determined the hyper parameter C (range 10^{-6} to 10^{2}) by 5-fold cross validation. Results of the per-class SVMs were averaged, resulting in one average decoding accuracy per DNN and image dataset. As expected, the untrained, noise and unecological DNNs performed at chance, whereas the object and scene DNNs had high performance similar to benchmark performance reported previously on object and scene categorization[16] (DNN for object ('ImageNet-CNN') classification on SUN-397 = 42.61 and Caltech-101 = 87.22; DNN for scene classification ('places-CNN') on SUN 397 = 54.42 and Caltech-101 = 65.18; chance level is 1/397=0.25% for SUN-397 and 1/101=0.99% for Caltech-101). Note that DNN performance depended on classified image material: on the scene image dataset (SUN-397) the scene DNN performed better than the deep object network, and vice versa for the object image dataset (Caltech-101).

Supplementary Table 2

	**Onset latency (ms)**	**Peak latency (ms)**
**Layer 1**	74(63-79)	130 (108-134)
**Layer 2**	74 (60-78)	120 (104-128)
**Layer 3**	69 (54-81)	110 (106-221)
**Layer 4**	66 (54-83)	109 (105-194)
**Layer 5**	63 (50-80)	108 (105-240)
**Layer 6**	63 (50-83)	134 (115-246)
**Layer 7**	76 (61-83)	172(152-216)
**Layer 8**	145 (129-164)	172 (169-304)

Supplementary Table 2 Onset and peak latencies for layer-wise MEG-DNN correlations for the object DNN (n = 15, 95% confidence intervals were determined by 1,000 bootstrap samples from the participant pool).

Supplementary Table 3

	Onset latency (ms)	Peak latency (ms)
**a)**		
**Object DNN**	74 (63-81)	118 (107-203)
**Scene DNN**	78 (62-85)	187 (98-223)
**Untrained DNN**	63 (57-64)	107 (102-134)
**Unecological DNN**	583 (147-604)	600 (76-709)
**Noise DNN**	74 (60-338)	109 (80-590)
**b) Object DNN minus**		
**Scene DNN**	130 (72-939)*	147 (36-632)*
**Untrained DNN**	166 (128-198)	203 (-74-231)
**Unecological DNN**	82 (78-85)	145 (108-224)
**Noise DNN**	88 (85-303)	226 (29-242)

Supplementary Table 3 Onset and peak latencies for time courses with which representations common between brain and DNNs emerged. **(a)** Onset and peak latencies in time courses of representational similarities between brain MEG signals and DNN layers. (**b)** Onset and peak latencies in the time course for the object DNN minus the time course of all other models for each model (*n* = 15, 95% confidence intervals were determined by 1,000 bootstrap samples from the participant pool). The scene DDN showed a significant difference only at a cluster threshold *P*-value of 0.05, and not when Bonferroni corrected by 4 for the number of comparisons (indicated by asterisk).

Supplementary Table 4

	V1		IT		IPS1&2	
	Spearman's R	*Significance (P-value)*	*Spearman's R*	*Significance (P-value)*	*Spearman's R*	*Significance (P-value)*
**a)**						
**Object DNN**	-0.65	0.003	0.50	0.007	0.48	0.005
**Scene DNN**	-0.68	0.002	0.26	0.155	0.30	0.08
**Untrained DNN**	-0.20	0.088	-0.47	0.002	-0.26	0.10
**Unecological DNN**	-0.26	0.003	-0.29	0.012	-0.08	0.42
**Noise DNN**	-0.40	0.001	-0.38	0.001	-0.03	0.77
**b) Comparison: Effect of object DNN minus effect of:**						
**Scene DNN**	0.12	0.136	0.39	0.005	0.47	0.002
**Untrained DNN**	-0.32	0.019	0.66	0.001	0.46	0.014
**Unecological DNN**	-0.48	0.001	0.65	0.001	0.44	0.001
**Noise DNN**	-0.42	0.001	0.64	0.001	0.40	0.002

Supplementary Table 4 Architecture, task constraints, and training procedure influenced the DNN's predictability of the position of brain regions in the visual hierarchy. **(a)** Correlations between layer number and fMRI-DNN representational similarities for the different models. **(b)** Comparison of object DNN against all other models (correlation computed after subtraction of corresponding fMRI-DNN representational similarities) (n = 15, significance determined by sign-permutation tests).

Supplementary Table 5

Layer	Conv1	Pool/ Norm 1	Conv 2	Pool/ Norm 2	Conv 3	Conv 4	Conv 5	Pool 5	FC1	FC2	FC3
**Units**	96	96	256	256	384	384	256	256	4096	4096	683/ 216
**Features**	55×55	27×27	27×27	13×13	13×13	13×13	13×13	6×6	1	1	1

Supplementary Table 5: Number of units and features for each DNN layer. Units and features of the DNN architecture were similar to those proposed in Krizhevsky et al., (2012) [29]**.** All DNNs were identical with the exception of the number of nodes in the last layer (output layer) as dictated by the number of training categories, i.e. 683 for the object DNN, 216 for the scene DNN, and 1000 for the untrained, unecological, and noise DNN. Abbreviations: Conv = Convolutional layer, Pool = Pooling layer; Norm = Normalization layer; FC1-3 = fully connected layers 1-3 (noted as layers 6-8 in entire architecture).

Supplementary Movie 1

Layer-wise representational similarity between human brains and the object DNN as determined by a surface-based searchlight analysis. The movie shows representational similarity ($P < 0.05$ cluster definition threshold, $P < 0.05$ cluster threshold) between fMRI-pattern derived RDMs and layer-specific RDMs for the object DNN from different angles.

Supplementary Text 1

We used a volumetric searchlight based analysis to provide an alternate view of data combined with a stricter statistical procedure that permits voxel-wise interest. Note that although a surface-based analysis provides higher specificity [53], it also uses part of available data by choosing only a single layer of voxels for the analysis. In contrast, a volumetric analysis may make use of all data present in local populations of voxels, such as when two voxels span the cortical sheet.

As for the surface-based analysis, for each subject we constructed an fMRI RDM for every voxel in the brain (4-voxel radius) based on the voxel's local activity patterns. We then correlated each voxel's RDM with the layer-specific DNN RDM (Spearman's R), yielding a 3D map of similarity for each layer. To compare results in a common framework, the resulting similarity maps were normalized into MNI space.

In line with the surface based analysis, similarities between the brain and the object DNN were largely confined to the dorsal and ventral streams (Suppl. Fig. 2, $P < 0.05$, FDR corrected). Low DNN layers had common representations prevalent in the occipital lobe of the brain, whereas higher DNN layers extended far into the temporal and parietal cortex. In particular, we found evidence for similar representations between brain representations and layer 8 reaching far into inferior parietal cortex bilaterally.

In sum, these results corroborate the surface-based analysis with an independent analysis approach and a statistic that allows for voxel-based inference.
